# The *Drosophila Gr28bD* product is a non-specific cation channel that can be used as a novel thermogenetic tool

**DOI:** 10.1038/s41598-017-19065-4

**Published:** 2018-01-17

**Authors:** Aditi Mishra, Autoosa Salari, Benton R. Berigan, Kayla C. Miguel, Marzie Amirshenava, Abbey Robinson, Benjamin C. Zars, Jenna L. Lin, Lorin S. Milescu, Mirela Milescu, Troy Zars

**Affiliations:** Division of Biological Sciences, University of Missouri, Columbia, MO 65211 USA

## Abstract

Extrinsic control of single neurons and neuronal populations is a powerful approach for understanding how neural circuits function. Adding new thermogenetic tools to existing optogenetic and other forms of intervention will increase the complexity of questions that can be addressed. A good candidate for developing new thermogenetic tools is the *Drosophila* gustatory receptor family, which has been implicated in high-temperature avoidance behavior. We examined the five members of the *Gr28b* gene cluster for temperature-dependent properties via three approaches: biophysical characterization in *Xenopus* oocytes, functional calcium imaging in *Drosophila* motor neurons, and behavioral assays in adult *Drosophila*. Our results show that *Gr28bD* expression in *Xenopus* oocytes produces a non-specific cationic current that is activated by elevated temperatures. This current is non-inactivating and non-voltage dependent. When expressed in *Drosophila* motor neurons, *Gr28bD* can be used to change the firing pattern of individual cells in a temperature-dependent fashion. Finally, we show that pan-neuronal or motor neuron expression of *Gr28bD* can be used to alter fruit fly behavior with elevated temperatures. Together, these results validate the potential of the *Gr28bD* gene as a founding member of a new class of thermogenetic tools.

## Introduction

Extrinsic control of cellular activity is a powerful paradigm for understanding how neural circuits regulate behavior. The standard approach relies on optogenetics to activate or inhibit neuronal activity with light^1,2^. However, the existing molecular tools used in optogenetic experiments have overlapping spectral sensitivities, limiting the number of neural components that can be controlled independently. As the field moves toward testing more and more components within a neural circuit, other modalities for extrinsic control must be identified. Thermogenetic tools use temperature as the physical controlling agent and provide an ideal complementary approach to optogenetics^1,3^.

Multiple classes of temperature-sensing proteins have been identified in *Drosophila*, including the transient receptor potential (TRP), ionotropic receptor, and gustatory receptor (Gr) families^4–8^. Each one of these classes provides unique opportunities for developing new thermogenetic tools, given their specific temperature sensitivity, ionic selectivity, kinetics, and regulation^1,8,9^. With a response threshold of about 25 °C and abrupt activation with temperature^6,10^, the TRP protein family (e.g., TRPA1) has been the most extensively studied and applied to understand neural circuits in *Drosophila* and other organisms^11–13^.

Although their potential for thermogenetic applications has not been explicitly tested so far, the *Drosophila* Grs are promising new candidates. This large family contains some members with ion channel activity and with advantageous temperature responses that fall within the physiological range of model organisms^7,14^. For example, the *Gr28bD* gene product has a neurophysiological response threshold similar to TRPA1, when tested in adult *Drosophila* neurons^7^. However, the Grs differ widely from TRPs in their primary structure and predicted molecular architecture, giving us an opportunity to discover new molecular mechanisms of temperature responsiveness and to engineer new tools for extrinsic control via temperature.

We examined here the temperature response of the five members of the Gr28b family (Gr28bA through Gr28bE) via three approaches: biophysical characterization in *Xenopus laevis* oocytes, functional calcium imaging in *Drosophila* motor neurons, and behavioral assays in adult *Drosophila*. Heterologous expression systems (e.g., oocytes or mammalian cell lines) are ideally suited for biophysical analysis, as any current can be better isolated and studied. Despite successful characterization of related gustatory and olfactory receptors in various artificial expression systems^14,15^, previous attempts to heterologously express Gr28b proteins were reported ineffective^7^. However, we were able to establish a quick and robust expression system to study the Grs in *Xenopus* oocytes, and found that Gr28bD is the only member of the Gr28b family that generates a temperature-dependent current. When expressed in *Drosophila* motor neurons, Gr28bD can alter the firing patterns of individual cells as a function of temperature. In behaving flies, neuronal expression of Gr28bD leads to temperature-dependent changes in behavior. Altogether, these results validate the potential of the *Gr28bD* gene as a founding member of a new class of thermogenetic tools.

## Results

### Biophysical properties of GR28bD in *Xenopus* oocytes

We report here the first successful expression of Gr28bD in *Xenopus laevis* oocytes, using established procedures (see Methods). We examined the temperature dependence, ionic selectivity, and basic kinetic properties of Gr28bD, using the two-electrode voltage-clamp (TEVC) technique to record oocyte currents under different conditions of temperature, membrane potential, and ionic composition of the extracellular solution. The other members of the Gr28b family were also tested, but no currents were observed.

**Figure 1:**
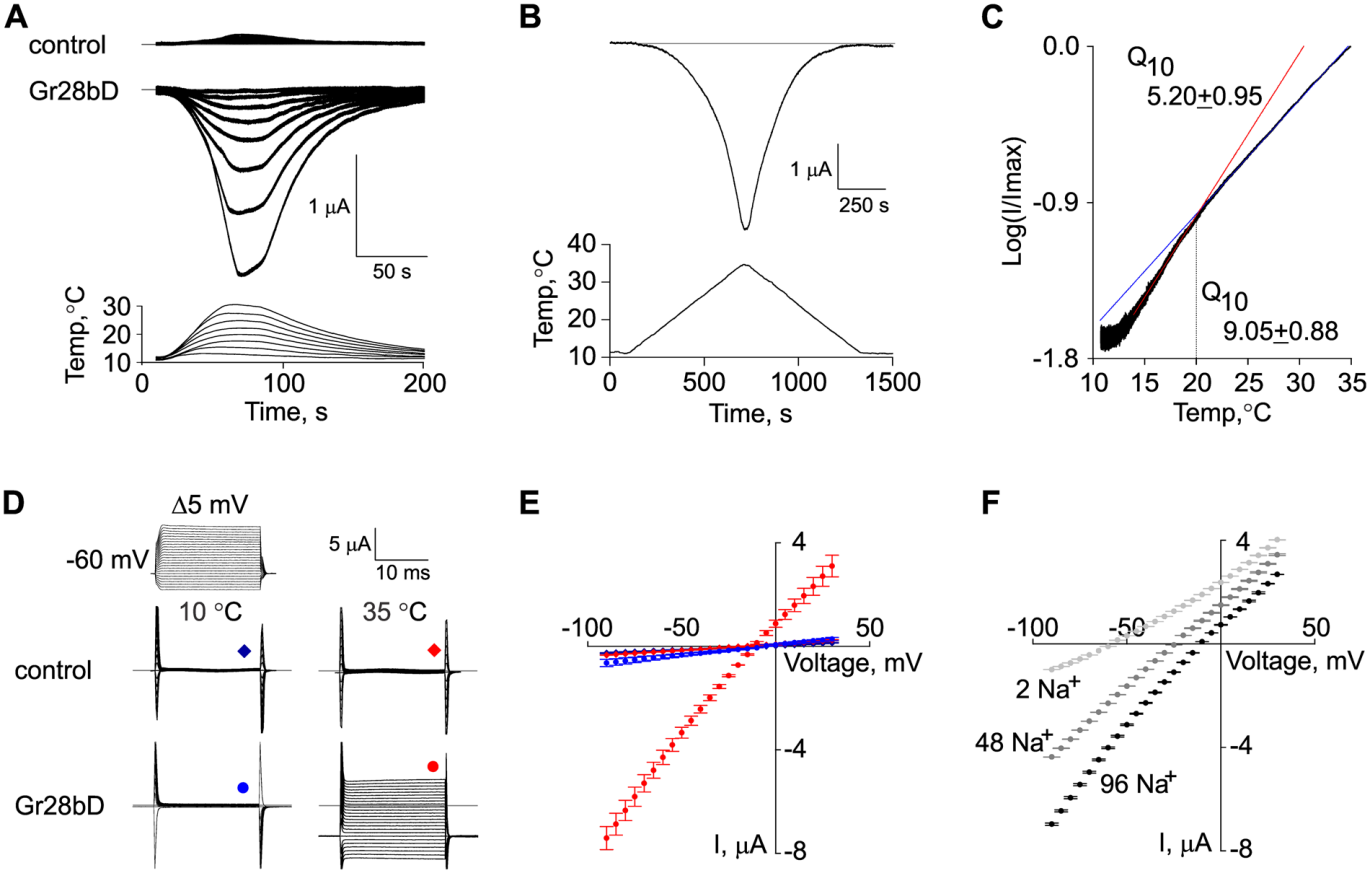
Biophysical properties of Gr28bD in *Xenopus* oocytes. (**A**) Temperature response: oocytes injected with *Gr28bD* cRNA display temperature-activated inward current in response to temperature steps. Control oocytes injected with RNAse-free water exhibit no significant current. (**B**) Response of Gr28bD current to slow temperature ramps (≈2.5 °C/60 s). (**C**) Temperature sensitivity: the logarithmic plot of normalized Gr28bD current versus slow ramp temperature exhibits two ranges of temperature sensitivity. Red and blue lines represent linear fits to data in each range (14–19 °C and 21–35 °C). Q_10_ values were calculated using the equation Q_10_ = 10^10×*s*^, where *s* is the slope of the linear fit. The average estimates are shown (n = 12). (**D**) Voltage response: control and Gr28bD currents in response to voltage steps at two different temperatures. Endogenous currents are small at all voltages and temperatures. (**E**) Current-voltage relationships for GR28bD-expressing and control oocytes at 10 and 35 °C, as obtained with the voltage step protocol in **D**. Gr28bD current does not show voltage sensitivity at the tested temperatures. The symbols are as in **D**. (**F**) Ionic selectivity: Gr28bD currents were obtained as in **E**, at 35 °C, with Na^+^ partially replaced by NMDG^+^ in the external solution (96 mM Na^+^ : 0 mM NMDG^+^, 48 : 48, and 2 : 94). The measured reversal potentials were (in mV): −12.0 ± 0.7, −23.7 ± 0.2, and −59.0 ± 0.9, respectively. The holding voltage was −60 mV in all experiments. Data shown in **A**, **B**, **C**, and **D** are representative examples. In **E** and **F**, data points are mean ± SEM (n = 6–8). The currents were obtained under ND96 solution (see Methods), except where otherwise noted.

When subjected to temperature steps (Fig. 1A, bottom panel), oocytes injected with *Gr28bD* cRNA exhibit large, temperature-sensitive currents (Fig. 1A, middle panel). In contrast, water-injected oocytes exhibit only a tiny amount of background current, with no significant temperature dependence (Fig. 1A, top panel). As can be observed from the example in Fig. 1A, our temperature application system cannot change temperature rapidly, which makes it difficult to estimate the kinetics of the Gr28bD current in response to a change in temperature. Cross-correlation analysis indicates that the current lags behind the temperature by approximately five seconds. Whether this lag is an experimental artifact or genuine channel kinetics remains to be determined through more precise temperature application experiments. For thermogenetic applications, it is very important to know whether the temperature-induced current can be maintained over long durations. From the response to temperature steps (Fig. 1A), we determined that the Gr28bD currents do not inactivate significantly over a 100-second time course. Moreover, the currents return to their initial value upon cooling.

The properties of a temperature-dependent current can be summarized by three quantities: the threshold of activation, the steepness of the response, and the saturation. To examine these properties, we subjected the oocytes to slow temperature ramps (approximately 2.5 °C/60 s), which should allow the channel to reach a state of quasi-equilibrium with respect to slow activation or inactivation kinetics, if any, and would also minimize temperature distribution artifacts within the recording chamber. The temperature was limited to a range of 10 –40 °C to avoid damage to the oocyte and to maintain reliable recording conditions. An example of the current elicited by a temperature ramp is shown in Fig. 1B. In this case, the cross-correlation analysis indicates a lag of less than one second. Furthermore, the on-ramp and the off-ramp produce approximately symmetrical responses, altogether confirming that channels are at quasi-equilibrium at these slow ramp rates. The logarithm of the normalized current versus temperature is shown in Fig. 1C. Inspection of the current response reveals a threshold of activation of approximately 14 °C. Above this temperature, the logarithm of the current increases linearly with a slope that corresponds to a Q_10_ of 9.05 ± 0.88, until 20 °C, where Q_10_ changes to 5.20 ± 0.95 (n = 12). These results were obtained with an extracellular solution (ND96) commonly used for oocyte recordings, containing 1.8 mM Ca^2+^. However, oocytes are known to express endogenous calcium-activated chloride currents^16^, which could contaminate our measurements, should the Gr channel have a significant calcium conductance. We tested this possibility using a nominally calcium-free ND96 recording solution^17^ and found Q_10_ values of 9.9 ± 1.3 and 5.6 ± 1.2 (n = 6), below and above 20 °C, respectively. These values are not statistically different from those reported above, obtained with 1.8 mM Ca^2+^ (unpaired t-test, p-value > 0.12).

The Gr28bD currents do not exhibit any detectable voltage dependence, at either low (10 °C) or high (35 °C) temperature, as illustrated in Fig. 1D and E. In control oocytes, the endogenous currents are very small across a broad voltage range (−100 mV to +30 mV), with no discernable voltage dependence. This implies that the currents recorded from *Gr28bD*-injected oocytes are generated by Gr28bD, with very little contamination from endogenous currents across a wide range of temperatures and membrane potentials.

Under the ND96 extracellular solution, containing (in mM) 96 NaCl, 2 KCl, and 1.8 CaCl_2_, the Gr28bD currents are inward at −60 mV (see Fig. 1A), with a measured reversal potential of −12 ± 0.7 mV (Fig. 1E). This value suggests that Gr28bD would act as an excitatory agent in thermogenetic applications. Nevertheless, to be able to predict the response of the channel and the nature of its effect in other biological preparations and solutions, it is necessary to determine its ionic selectivity. Under the ND96 solution and assuming 7 mM Na^+^, 100 mM K^+^, and 0.1 mM Ca^2+^ intracellular concentrations^18^, the predicted Nernst potentials would be approximately +69 mV for Na^+^, −104 mV for K^+^, and +24 mV for Ca^2+^, at 35 °C. The measured reversal potential of −12 mV under ND96 does not match any of these values, indicating that the channel is conducting more than one type of ion. Considering these measured and predicted values, the relative K^+^ conductance must be at least 35%, as would be calculated assuming zero relative Na^+^ conductance, or as much as 47%, assuming zero relative Ca^2+^ conductance.

To further investigate the relative conductance for Na^+^ ions, we recorded Gr28bD currents under various Na^+^ concentrations, with Na^+^ ions accordingly replaced by NMDG^+^, which is known not to permeate through Na^+^ or cation non-selective channels^19^. The relative conductance for an ion can be calculated from the ratio between the observed shift in the reversal potential of the current and the predicted shift in the Nernst potential for that ion. As illustrated in Fig. 1F, when the extracellular Na^+^ concentration is reduced from 96 to 48 mM, the measured reversal potential changes from −12 to −23.7 ± 0.17 mV, while the predicted Na^+^ Nernst potential changes from +69 to +51 mV, corresponding to an estimated 65% relative Na^+^ conductance. When the extracellular Na^+^ is reduced to 2 mM, the measured reversal potential changes to −59 ± 0.9 mV, while the predicted Na^+^ Nernst potential changes to −33 mV, corresponding to an estimated 45% relative Na^+^ conductance.

These results average to a 55% relative Na^+^ conductance. Considering this and the above estimates for the K^+^ conductance (35% to 47%), the partial Ca^2+^ conductance should be less than 10%. We also tested low-calcium extracellular solutions, one nominally Ca^2+^- free and one with 0.6 mM Ca^2+^, and found only a small negative shift in the reversal potential of less than 1 mV. This result is consistent with a Ca^2+^ conductance of less than 4%. Additional experiments in which Cl^−^ was replaced with gluconate (C_6_H_11_O_7_^−^) did not alter the reversal potential, suggesting that the Gr28bD current is neither carried by Cl^−^ ions nor contaminated by endogenous chloride currents (data not shown).

Altogether, these results clearly indicate the non-selective cationic nature of the current, similar to that of several TRP channels^20^. Although further experiments are necessary to obtain more precise values, most of the Gr28bD conductance appears to be due to Na^+^ (55%) and K^+^ (35–47%), with any potential remainder due to Ca^2+^ ions. In contrast to Gr28bD, expression of the four other Gr28b isoforms (A, B, C, and E) did not produce any measurable current, temperature-sensitive or not, above the endogenous level. Moreover, co-expression of all possible binary combinations of these isoforms did not elicit any temperature-dependent current (data not shown). However, the possibility of functional Gr28b heteromers cannot be ruled out, as more than two isoforms may be needed to form a functional protein.

### Modulation of neuronal activity by *Gr28bD*

To use a thermogenetic tool, one must have a good way to calibrate and monitor its effects on neuronal activity. As shown by our biophysical experiments and by a previous study^7^, Gr28bD generates a temperature-sensitive non-selective cation current in both *Xenopus* oocytes and *Drosophila* neurons, and elicits strong proboscis extension when expressed in sweet-responsive fruit fly chemosensors. According to these results, Gr28bD should, in principle, generate excitatory inward currents in target neurons. However, enough current must be generated relative to the battery of endogenous currents normally expressed by a cell to modulate cellular activity. Electrophysiology is potentially the most precise measuring method^7^, but it is not suitable for large-scale monitoring of neuronal populations. Therefore, we used imaging techniques to test Gr28bD in adult *Drosophila*, in combination with a genetically-encoded calcium indicator (GCaMP6f), to determine if it produces a robust, temperature-dependent effect that can be easily identified, analyzed, and interpreted with common imaging and data analysis techniques. We also tested control flies expressing only GCaMP6f.

**Figure 2:**
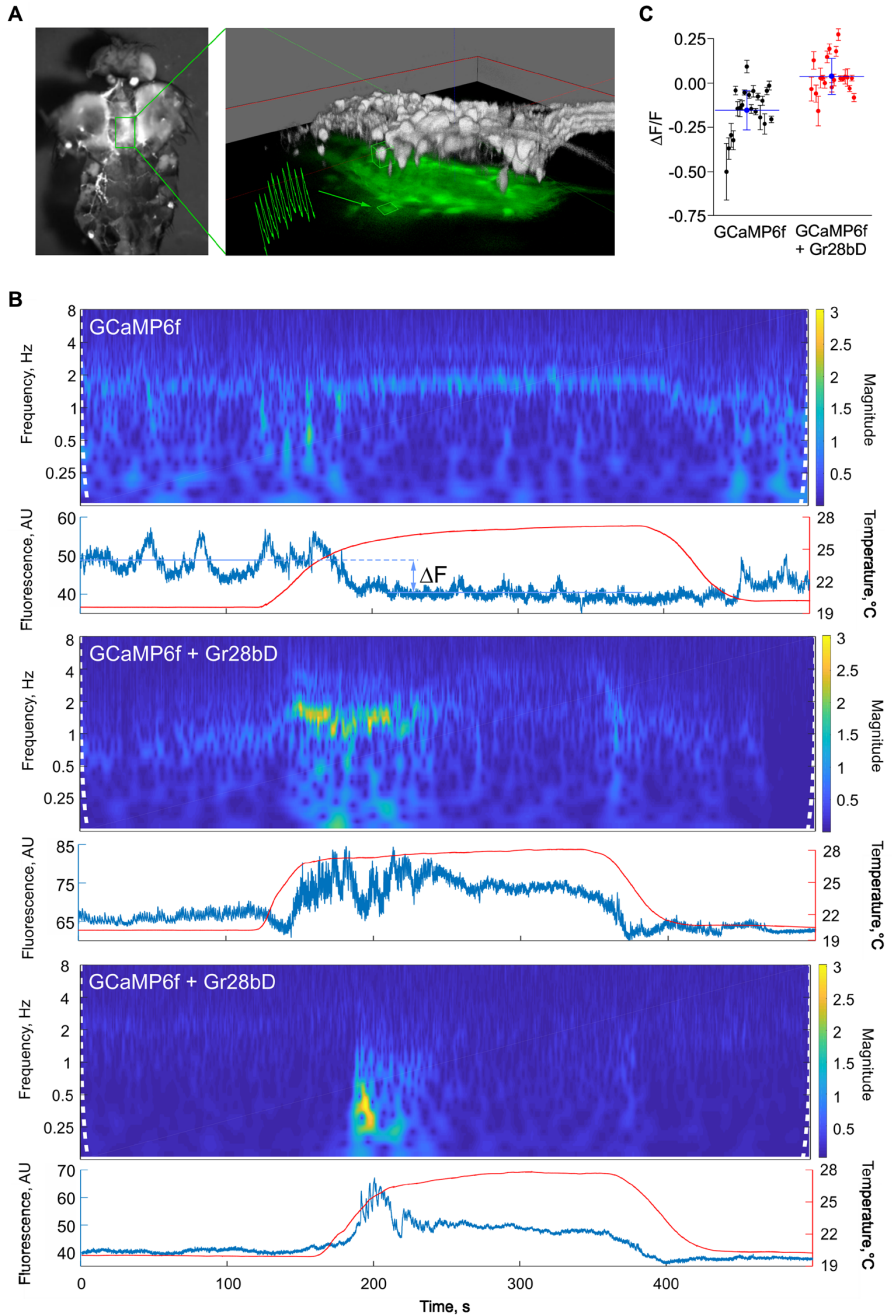
Temperature-dependent modulation of cellular activity by Gr28bD. (**A**) Left panel: dorsal view of adult *Drosophila* dissected to expose ventral nerve cord motor neurons to saline bath. Right panel: composite image of epifluorescence (green on black) and two-photon 3D reconstruction (gray scale) of abdominal neuromere neurons expressing GCaMP6f. The green trace is an example of single-cell activity, as obtained via epifluorescence from the indicated region of interest. (**B**) Single-cell fluorescence signals (blue traces) obtained in response to a temperature step (red traces), analyzed by continuous wavelet-transform (spectrogram). Top panel: representative data from control flies expressing only GCaMP6f in motor neurons. Middle and bottom panels: representative data sets illustrating two types of temperature responses in flies expressing GCaMP6f + Gr28bD in motor neurons. (**C**) Change in average fluorescence between low and high temperature. ΔF/F was calculated as (F_H_−F_L_)/F_L_, where F_L_ and F_H_ are the average fluorescence intensities (in arbitrary units) for a given cell, at low and high temperature. Data points are mean ± SDV (n = 21 cells from five flies for GCaMP6f, and n = 20 cells from six flies for GCaMP6f + Gr28bD). The blue lines represent mean ± SDV for each data set. ΔF/F = −0.1524 ± 0.1358 in control flies and 0.03656 ± 0.10 in GR28bD-expressing flies (unpaired t-test; p-value < 0.00001).

For these tests, we focused on the motor neurons in the lateral abdominal neuromeres, located in the ventral nerve cord (VNC). As illustrated in Fig. 2A, these neurons are a good target for calcium imaging experiments, as they are easy to identify and consistently exhibit robust firing activity. Moreover, these neurons are conveniently located in the same plane and exhibit calcium fluorescence with comparable intensity and good signal-to-noise ratio, altogether making it easy to image multiple cells at the same time. Additionally, motor neurons are generally larger than other neuronal types, making them a good test system to determine whether expression of Gr28bD can generate enough current to modulate cellular activity.

The thermosensitive effect of Gr28bD on neuronal activity was tested with temperature steps alternating between 19 and 28 °C. We recorded from 21 GCaMP6f control cells in five flies and 20 Gr28bD + GCaMP6f cells in six GR28bD-expressing flies. Under repetitive application of temperature steps, calcium fluorescence activity lasted for 73.6 ± 4.5 minutes in control flies and 49.7 ± 7.4 minutes in GR28bD-expressing flies. One would anticipate that changing temperature by approximately 10 °C would have an effect on neuronal activity, even in control flies. Would changes be different between control and Gr28bD-expressing flies? Indeed, as illustrated in Fig. 2B, control flies (top panel) and Gr28bD-expressing flies (middle and bottom panels) respond differently to temperatures steps. The most notable distinction is in how the average fluorescence intensity changes between low and high temperature (Fig. 2C). In control flies, the average fluorescence level is attenuated by high temperature (ΔF/F = −0.1524 ± 0.1358), but it is slightly increased in Gr28bD-expressing flies (ΔF/F = 0.03656 ± 0.10) (unpaired t-test; p-value < 0.00001).

Although the temperature-induced change in average fluorescence in Gr28bD-expressing neurons is small (≈3.7%) it should be regarded as relative to the control group. Accordingly, the Gr28bD-specific effect is an overall increase in fluorescence of almost 20%. The average calcium fluorescence is proportional to the overall neuronal activity^21,22^, independent, to some degree, of the specific firing pattern (e.g., tonic spiking vs. bursting). Therefore, the observed changes in average fluorescence elicited by high temperature can be interpreted as a slow-down of neuronal activity in control flies versus an increase in activity in Gr28bD-expressing flies.

GCaMP6f is in principle capable of reporting individual action potentials. However, without simultaneous electrical recordings, it is difficult to determine whether the individual calcium fluorescence events detected in our data correspond to single action potentials or to more complex patterns of activity (e.g., episodes of high-frequency firing). Thus, we used the continuous wavelet-transform (CWT) with a Morlet wavelet^23^ to examine the spectral content of the calcium fluorescence data. As illustrated by the example recordings in Fig. 2B, there are significant differences between control (top panel) and Gr28bD-expressing flies (middle and bottom panels).

In control neurons, the spectral content of fluorescence data changes relatively little with temperature, with respect to both frequency and amplitude. These neurons are active at both low and high temperatures, as demonstrated by the multiple spectral bands that are visible in the 1–4 Hz frequency range, some of which potentially corresponding to the repetition rate of action potentials. The strongest of these bands (1–2 Hz) becomes slightly more intense and more regular at high temperature. In contrast, some of the Gr28bD-expressing neurons exhibit similar tonic activity at 19 °C (n = 8), while others are in a more quiescent state (n = 12). The onset of high temperature triggers a remarkable increase in the amplitude of high-frequency oscillations, lasting as much as 100 seconds. The effects of temperature were reversible in both control and Gr28bD-expressing flies. After each four-minute exposure to 28 °C, the cells resumed their normal activity when temperature was returned to 19 °C, throughout the lifetime of the preparation.

### Activation of Gr28bD alters behavior

**Figure 3:**
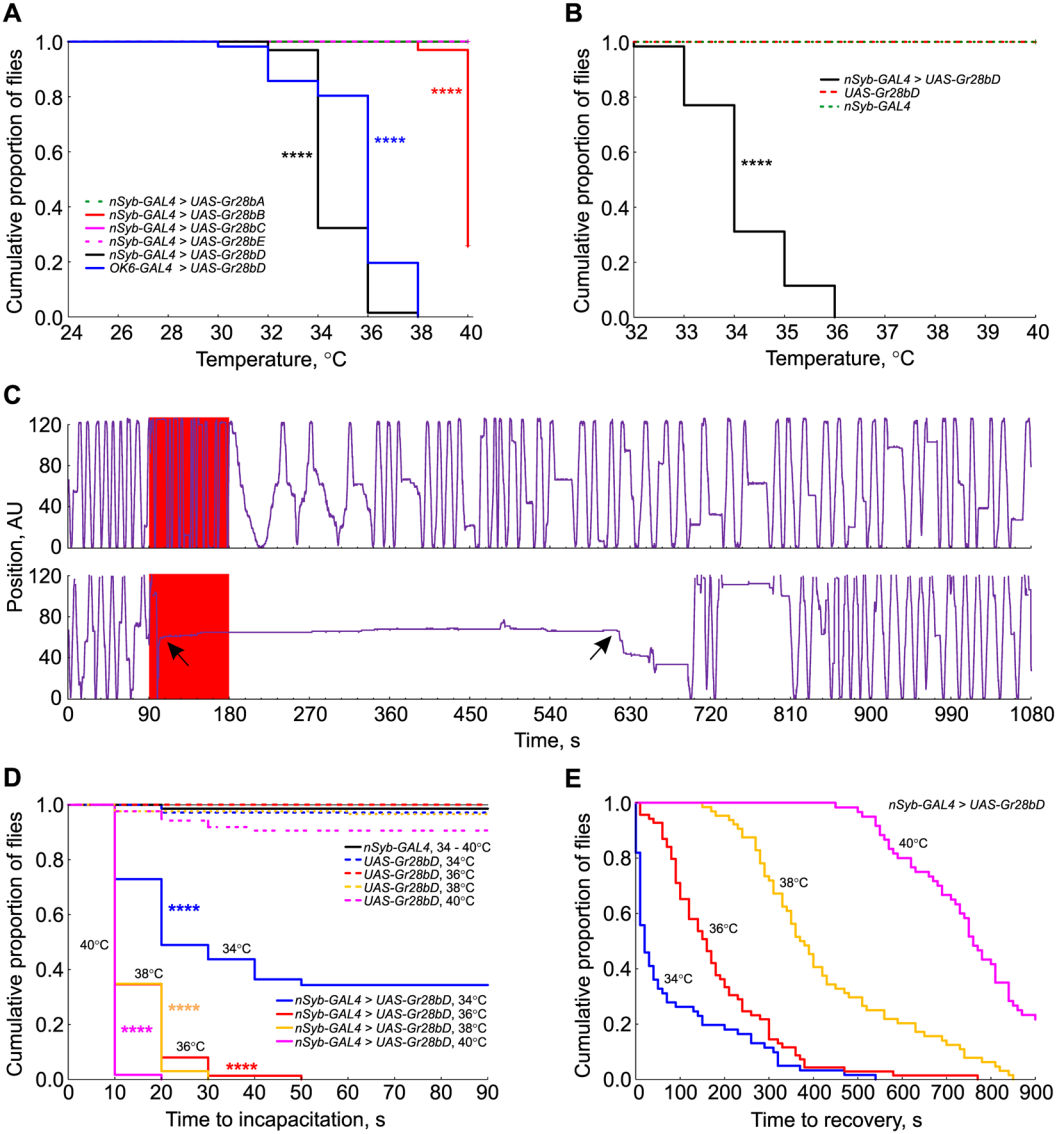
Temperature-dependent modulation of fly behavior by Gr28bD. (**A**) Incapacitation by temperature: flies overexpressing *GR28b* genes had their locomotor activity recorded for 90 seconds at 24–40 °C in two-degree steps. The plot shows the cumulative proportion of flies not incapacitated at each temperature. Most flies with pan-neuronal or motor neuron overexpression of GR28bD were temporarily incapacitated at 34 or 36 °C, respectively. Gr28bB overexpression had an effect at the very high end of the tested temperature range. (**B**) Control and *Gr28bD*-overexpressing flies were exposed to 32–40 °C in one-degree steps. Most flies overexpressing *Gr28bD* pan-neuronally were incapacitated at 33–35 °C. The heterozygous genetic control flies did not show incapacitation over the tested temperatures. Incapacitation is faster and recovery is slower at higher exposure temperatures. (**C**) Example position traces of a non-incapacitated fly (top panel) and of an incapacitated fly that recovered (bottom trace). The red area represents the exposure to high temperature and the arrows mark the times of incapacitation and recovery. (**D**) Time to incapacitation at different temperatures: flies overexpressing *Gr28bD* and flies heterozygous for the *GAL4* driver and *UAS*-*Gr28bD* construct were tested. Most flies with *Gr28bD* pan-neuronal overexpression were incapacitated within 10 seconds at 36, 38, and 40 °C, but it took much longer at 34 °C. Few flies from the control genotypes were incapacitated at the temperatures tested. (**E**) Time to recovery from incapacitation: flies recovered faster when subjected to lower temperature. In **A**, **B**, and **D**, statistical difference between control and experimental genotypes was determined with a Kaplan-Meier test, p < 0.000001 = ****. In each experiment, 60–80 flies of each genotype were tested.

Our imaging experiments demonstrate the ability of Gr28bD to induce temperature-dependent effects in the firing activity of individual neurons. However, we also see temperature effects in control flies. To determine how these specific and non-specific effects scale up to the level of the entire organism, we investigated the behavioral response of flies to different temperatures in the 24–40 °C range. *Gr28bD* and each of the other four different transgenes in the *Gr28b* family were individually tested with pan-neuronal overexpression using the *nSyb*-*GAL4* driver. An initial screening exposed flies to temperatures ranging from 24 – 40 °C in two-degree steps (Fig. 3A). Most of the flies overexpressing *Gr28bD* became incapacitated at 34 °C, in contrast to flies overexpressing the *Gr28bA, C* and *E* transgenes, which did not incapacitate. A temperature response was also observed for *Gr28bB* flies, but only at the upper temperature limit of our experimental paradigm (40 °C), which prevented further investigation. We also tested the effect of a more restricted overexpression of *Gr28bD*, in motor neurons only, using the *OK6*-*GAL4* driver. In this case, we found that most of the flies were incapacitated at 36 °C (Fig. 3A). In the remainder of the Results section all references to overexpression should be read as “pan-neuronal” overexpression. To obtain a better resolution of the *Gr28bD*-induced effect, flies from control and experimental genotypes were subjected to temperatures in the 32–40 °C range in one-degree steps. Flies overexpressing *Gr28bD* became incapacitated between 33 and 36 °C, with most of the flies incapacitated at 34 °C (Fig. 3B). In contrast, no genetic control flies were incapacitated.

Next, we tested whether the time to incapacitation and the time to recovery from incapacitation are affected by the exposure temperature. We examined these two quantities by exposing flies overexpressing *Gr28bD* to a sudden increase in temperature for 90 seconds (Fig. 3C). All flies exposed to 36, 38, and 40 °C became incapacitated during the 90 second exposure, with more than 50% reaching incapacitation within 10 seconds (Fig. 3D). At 34 °C, only ≈65% of the exposed flies became incapacitated and the time to incapacitation was significantly longer, suggesting that 34 °C is a threshold value for temperature-sensitive behavior. In contrast, none of the genetic control flies were incapacitated within the 90 seconds exposures at any tested temperature, with the exception of the *UAS-Gr28bD* heterozygous flies. About 10% of these flies became incapacitated at 40 °C, potentially due to a leaky expression of the *UAS-Gr28bD* transgene. The time to recovery for flies overexpressing *Gr28bD* was more graded than the time to incapacitation (Fig. 3E). Flies exposed to high temperatures took an exponentially longer time to recover than flies exposed to low temperatures. From 34, 36, 38, and 40 °C, the 50% recovery time was 20, 162, 383, and 760 seconds, respectively. Moreover, at 40 °C, the recovery was not complete within the 1080 seconds of the recovery phase of the experiment with 22% of the flies remaining incapacitated.

## Discussion

The ability to control neuronal activity is the most direct and powerful approach for understanding how neural circuits control behavior. An ideal suite of tools for extrinsic neuronal control would include activating and inactivating methods that can target multiple cell types and neuronal populations in a behaving animal. The development of thermogenetic tools as a complement to optogenetics and other approaches is a promising step toward multi-modal control of neuronal activity^1,2^. The *Drosophila* gustatory receptor gene family, which has been largely unexplored with respect to temperature responsiveness, provides an opportunity to identify novel thermogenetic tools.

We show here that one member of the Gr28b family, Gr28bD, generates a temperature-dependent cation current when expressed in *Xenopus* oocytes. This current does not require any explicit co-expression of additional factors, suggesting that Gr28bD forms a previously uncharacterized temperature-sensitive ion channel (Fig. 1). Moreover, we show that overexpression of Gr28bD in motor neurons can be used to drive temperature-dependent neuronal activity (Fig. 2). The expression system is sufficient to drive activity in these relatively large neurons, suggesting that the *Gr28bD* product can be used in multiple cellular contexts. Finally, we show that temperature activation of Gr28bD overexpressed in neurons can have profound effects on behavior (Fig. 3). Expression of Gr28bD in all neurons gives rise to temperature-dependent paralysis, with the time to incapacitation and the recovery time both related to exposure temperatures. We also demonstrate that flies with Gr28bD expression restricted to motor neurons display a similar temperature-dependent paralysis, but shifted by approximately +2 °C. This small shift could be explained by a difference in Gr28bD expression levels, as caused by different strengths of the two *GAL4* drivers, and/or by differences between the neuronal types overexpressing Gr28bD. According to these results, the *Gr28bD* gene product is useful as a thermogenetic tool and is a good starting point for designing new molecular tools for extrinsic control of neuronal activity.

Our first attempts to demonstrate that behavior can be altered with Gr28bD activation showed that the gene product has a sharp temperature effect, with nearly all flies reaching paralysis within a narrow temperature window (33–36 °C). This temperature range for Gr28bD is about 4 °C higher than that required to activate and paralyze TRPA1-expressing flies (data not shown). Interestingly, the time required for recovery from paralysis was temperature dependent, with flies exposed to higher temperatures taking longer to recover from paralysis. The difference in recovery at different temperatures could be a function of the *Gr28bD* product or of the neural systems that need to recover from extrinsic activation. However, the temperature-sensitive currents recorded in *Xenopus* oocytes expressing Gr28bD follow the change in temperature equally rapidly (Fig. 1A), within seconds, between any two temperatures. This suggests that the temperature-dependent slow recovery rates observed in the behavioral experiments are the result of neuronal properties, rather than Gr28bD temperature sensitivity and kinetics.

Further experiments are necessary to more precisely determine the time response of the Gr28bD to changes in temperature. With the estimates we have so far (seconds), it is possible that Gr28bD kinetics are slow compared to fly behaviors such as the escape response or the tuning of wing movements in flight^24,25^. Nevertheless, the response kinetics are compatible with the testing of other behaviors, such as phototaxis, negative gravitaxis, and conditioning, which are typically measured in the seconds range^26–28^.

While Gr28bD has clear temperature sensitive effects that can be corroborated at all tested levels (oocytes, individual neurons, and whole organism), the other members of the Gr28b family have no observable effect (Gr28bA and C) or are less consistent across experimental paradigms (Gr28bB and E). Expression of Gr28bB in *Xenopus* oocytes does not show a temperature-dependent current (data not shown). However, pan-neuronal expression of Gr28bB gives rise to temperature-dependent paralysis at the highest probed temperature of 40 °C (Fig. 3A). One possible explanation is that Gr28bB requires higher temperatures to activate and generate current, temperatures which could not be reached in the *Xenopus* experiments. Alternatively, there may be insufficient expression of Gr28bB in *Xenopus* oocytes, causing a lack of detectable current. Heterologous expression systems that can tolerate higher temperatures may be able to address this issue. Nevertheless, the observed temperature-dependent paralysis is an encouraging result, suggesting that the *Gr28bB* gene product may also generate a temperature responsive current. Finally, there is evidence from a previous study that Gr28bE has temperature response properties when expressed in the hot-cells of the *Drosophila* antennae^7^. In our experiments, expression in *Xenopus* oocytes and in all *Drosophila* neurons does not show any detectable temperature-dependent effect of Gr28bE. There may be factors in the hot-cells that provide the environment for Gr28bE temperature responsiveness that are lacking in other neurons and in *Xenopus* oocytes.

To summarize, we show that Gr28bD, a member of the *Drosophila* gustatory receptor family, can be used as a thermogenetic tool. Gr28bD produces a temperature-dependent current in heterologous cells, is able to activate multiple cell types in the fly, and can be used to interrogate neural circuit function in imaging and behavioral studies. Further testing of the gustatory receptor family may identify new members with temperature sensitive properties that may aid in the development of designer thermogenetic tools. To be useful in multiple model systems and cellular contexts, these proteins could be further engineered with respect to their temperature sensitivity (activation threshold and Q_10_), ionic selectivity, regulation by other physical and chemical factors, and cellular localization.

## Methods

All reagents were purchased from Sigma-Aldrich, USA (St. Louis, MO).

### *Gr28b* constructs and oocyte expression

The *Gr28b* genes were cloned in pcDNA3.1+/C-(K)DYK (GenScript). Complementary RNA (cRNA) was synthesized using T7 polymerase (mMessage mMachine kit, Ambion, USA), after linearizing the DNA with XmaI. All *Gr28b* constructs were expressed in *Xenopus laevis* oocytes, and studied 1–3 days after cRNA injection. The oocytes were incubated at 15 °C in an ND96 solution containing (in mM): 96 NaCl, 2 KCl, 5 HEPES, 1 MgCl_2_, 1.8 CaCl_2_, and 50 μg/ml gentamicin, adjusted to pH 7 with NaOH^29^.

### Flies and rearing conditions

The *UAS-Gr28b A, B, C, D*, and *E* lines were generously provided by Paul Garrity, Brandeis University^7^. The *nSyb*-*GAL4*, *OK6-GAL4*, and *UAS-GCaMP6f* lines were obtained from the Bloomington Stock Center, Indiana. Flies were created to overexpress *UAS-Gr28b* transgenes with the pan-neuronal *nSyb-GAL4* driver and *UAS-Gr28bD* with the motor neuron *OK6-GAL4* driver. Flies heterozygous for *nSyb-GAL4* or *UAS-Gr28b* transgenes were used as negative genetic controls. Flies expressing *UAS-Gr28bD* + *UAS-GCaMP6f*, or heterozygous *UAS-GCaMP6f* (control), with the *OK6-GAL4* driver, were created for the calcium fluorescence imaging experiments. Flies were reared on cornmeal-yeast medium at 25 °C in a 12 hour light-dark cycle at 60% relative humidity. Flies used for thermo-tolerance tests were 2–10 days of age.

### Oocyte electrophysiology and data analysis

After incubation, the oocytes were transferred into a 150 μl recording chamber and examined via the two-electrode voltage-clamp recording technique (OC-725C amplifier, Warner Instruments, USA). Oocyte currents were low-pass filtered at 5 kHz and digitized at 10 kHz using a Digidata 1440a data acquisition system and pClamp 10 software (Molecular Devices, USA). Microelectrode resistance was 0.3–1 MΩ when filled with 3 M KCl. Several external recording solutions were used, all based on the standard ND96 recipe, containing (in mM): 96 NaCl, 2 KCl, 5 HEPES, 1 MgCl_2_, 1.8 CaCl_2_, pH 7.6 with NaOH. In some experiments, a nominally Ca^2+^-free (0 mM CaCl_2_) and a low Ca^2+^ (0.6 mM CaCl_2_) ND96 solutions were used. A low-Cl^−^ solution was formulated as: 96 NaC_6_H_11_O_7_ (sodium gluconate), 2 KC_6_H_11_O_7_ (potassium gluconate), 5 HEPES, 1 MgCl_2_, 1.8 CaCl_2_, pH 7.6 with NaOH. Two low-Na^+^ solutions were made, one with 48 mM and one with 2 mM Na^+^, obtained by substituting the corresponding amounts of NaCl with NMDG-HCl. All experiments were performed between 10 and 40 °C, with the temperature regulated by a bipolar temperature controller (CL-100, Harvard Apparatus, USA) and a dual in-line heater/cooler (SC-20, Warner Instruments, USA). Q_10_ values were calculated from slow temperature ramp experiments, using the equation Q_10_ = 10^10×*s*^, where *s* is the slope of the linear fit to the logarithmic plot of the normalized current versus temperature. The equilibrium potentials were calculated using the Nernst equation: ${E}_{{\rm{X}}}=\frac{RT}{zF}\,\mathrm{ln}\,\frac{{[X]}_{O}}{{[X]}_{I}}$, where *E*_X_ is the equilibrium potential for a given ion X, of extracellular and intracellular concentrations [*X*]_O_ and [*X*]_I_, respectively. *R*, *T*, *z*, and *F* are the usual quantities: the gas constant, the temperature, the valence of the ion, and Faraday’s constant, respectively. The data were analyzed with Clampfit 10 (Molecular Devices, USA) and Origin 2017 (OriginLab, USA).

### Calcium fluorescence imaging and data analysis

Flies, 5–11 days old, were dissected as previously described^30^. To eliminate descending thermotaxis signals to the ventral nerve cord, the head of the fly was removed before imaging. The dissected preparations were perfused in external saline containing (in mM): 103 NaCl, 3 KCl, 5 N-tris(hydroxymethyl) methyl-2-aminoethane-sulfonic acid, 8 trehalose, 10 glucose, 26 NaHCO_3_, 1 NaH_2_PO_4_, 1.5 CaCl_2_, 4 MgCl_2_, with an osmolarity between 270–275 mOsm. The saline was continuously bubbled with 95% O_2_/5% CO_2_ to a pH of 7.3. Perfusate temperature was regulated with a bipolar temperature controller (CL-100, Harvard Apparatus, USA), a SC-20 dual in-line heater/cooler (Warner Instruments, USA), and a LCS-1 liquid cooling system (Warner Instruments, USA). A custom imaging stage was designed in SketchUp (sketchup.com) and 3D printed with PLA using an Ultimaker Original+ (Ultimaker, Netherlands). Lateral motor neurons in the abdominal neuromeres^31^ were imaged on a customized upright microscope (Scientifica, UK) equipped with epifluorescence and two-photon imaging. For wide-field illumination, CoolLED p100 LED sources (CoolLED Ltd., UK), GFP filters (Chroma, USA), and a 40X water-immersion objective (Olympus LUMPLFLN40XW) were used. Images were acquired using a Hamamatsu sCMOS Flash 4.0 V2 camera (Hamamatsu Photonics KK, Japan), typically with a 25 ms exposure time. For two-photon imaging, we used a Ti:Sapphire MaiTai HP laser (Newport, USA). The QuB program (MilescuLabs.biology.missouri.edu/QuB) was used for 3D data mapping, experiment control and visualization, and image acquisition^32^. ScanImage 3.8.1 (Vidrio Technologies, USA) was used to control the two-photon hardware. Fluorescence signals were digitally processed, analyzed with the QuB software, and the spectral content was calculated in MATLAB 2017a (MathWorks, USA) using the continuous wavelet-transform (CWT) and a Morlet wavelet.

### Behavioral experiments

Behavioral experiments were performed using the heat box paradigm as previously described^33,34^. In the heat box, flies were allowed to walk the length of a chamber (l = 3.4 cm, w = 3 mm, h = 1 mm) lined top and bottom with Peltier elements. A light sensor was used to detect the position of the fly. The temperature in the chamber was controlled using custom software^35^. Additional custom software was used to analyze the activity of flies. In the thermotolerance assays, the temperature of the chamber was initially increased in steps of 2 °C from 24  to 40 °C, and later in steps of one degree from 32  to 40 °C. The movement of each fly was measured at each temperature step for 90 seconds. Six or seven trials were conducted for each experimental genotype, with 10–12 flies in each trial. A fly was considered incapacitated if it was inactive for at least 45 s within a 90 s window. To determine the time-to-incapacitation and the time-to-recovery, flies were allowed to acclimate at 24 °C for 90 s prior to a temperature jump to 34 –40 °C for 90 s, and then allowed a recovery time of 15 minutes. The movement of flies was recorded for the entire duration of the test. Observations from incapacitation assays were analyzed with the Statistica 8.0 software (StatSoft, USA), using Kaplan-Meier survival analysis with p-values of less than 0.05 considered significant.

### Data availability

The datasets generated during the current study are available from the corresponding authors on reasonable request.
